# Rehabilitation of Aging Persons: The Past, The Present & The Promise

**DOI:** 10.1093/geroni/igaa057.3208

**Published:** 2020-12-16

**Authors:** Kenneth Ottenbacher

**Affiliations:** The University of Texas Medical Branch at Galveston, Galveston, Texas, United States

## Abstract

The 2020 Excellence in Rehabilitation of Aging Persons Award presentation will address my efforts over the past 35 years related to research methods, functional status, mobility, and self-care. Studies conducted in the past 25 years on disability and recovery in older adults with an emphasis on minority health will be presented. Research examining rehabilitation outcomes related to health care reform including the Improving Medicare Post-Acute Care Transformation (IMPACT) Act, and using Medicare files, will be described. The role of Data Science and Discovery, as defined by the NIH and related to rehabilitation in older adults, will also be presented.

